# Characteristics of Hospitalized Cases with Influenza A (H1N1)pdm09 Infection during First Winter Season of Post-Pandemic in China

**DOI:** 10.1371/journal.pone.0055016

**Published:** 2013-02-05

**Authors:** Cuiling Xu, A. Danielle Iuliano, Min Chen, Po-Yung Cheng, Tao Chen, Jinghong Shi, Jing Yang, Lijie Wang, Fan Yuan, Marc-Alain Widdowson, Yuelong Shu

**Affiliations:** 1 National Institute for Viral Infectious Disease Control and Prevention, Chinese Center for Disease Control and Prevention, Beijing, China; 2 Influenza Division, National Center for Immunization and Respiratory Diseases, Centers for Disease Control and Prevention, Atlanta, Georgia, United States of America; University of Hong Kong, Hong Kong

## Abstract

**Background:**

Influenza A (H1N1)pdm09 (2009 H1N1) re-circulated as the predominant virus from January through February 2011 in China. National surveillance of 2009 H1N1 as a notifiable disease was maintained to monitor potential changes in disease severity from the previous season.

**Methodology/Principal Findings:**

To describe the characteristics of hospitalized cases with 2009 H1N1 infection and analyze risk factors for severe illness during the 2010–2011winter season in China, we obtained surveillance data from hospitalized cases with 2009 H1N1 infection from November 2010 through May 2011, and reviewed medical records from 701 hospitalized cases. Age-standardized risk ratios were used to compare the age distribution of patients that were hospitalized and died due to 2009 H1N1 between the 2010–2011winter season to those during the 2009–2010 pandemic period. During the 2010–2011 winter season, children less than 5 years of age had the highest relative risk of hospitalization and death, followed by adults aged 65 years or older. Additionally, the relative risk of hospitalized cases aged 5–14 and 15–24 years was lower compared to children less than 5 years of age. During the winter season of 2010–2011, the proportions of adults aged 25 years or older for hospitalization and death were significantly higher than those during the 2009–2010 pandemic period. Being male, having a chronic medical condition, delayed hospital admission (≥3 days from onset) or delayed initiation of antiviral treatment (≥5 days from onset) were associated with severe illness among non-pregnant patients ≥2 years of age.

**Conclusions/Significance:**

We observed a change in high risk groups for hospitalization for 2009 H1N1 during the winter months immediately following the pandemic period compared to the high risk groups identified during the pandemic period. Our nationally notifiable disease surveillance system enabled us to understand the evolving epidemiology of 2009 H1N1 infection after the pandemic period.

## Introduction

On June 11, 2009, the World Health Organization (WHO) declared a global pandemic caused by a novel swine-origin influenza A(H1N1) virus [1]. By the end of the 2009 calendar year, most countries around the world had experienced at least one epidemic waves of influenza A (H1N1)pdm09 [2].

Although the WHO declared an end to the pandemic period on August 2, 2010, influenza A (H1N1)pdm09 (2009 H1N1) virus continued to circulate and became the most commonly detected virus in many northern hemisphere temperate countries in the winter season of 2010–2011 [3]–[7]. In some northern hemisphere countries, but not all, the impact of 2009 H1N1 in the 2010–2011 season was greater than in the previous year, most notably in the United Kingdom (UK) where intensive care units were stressed by large numbers of patients requiring ventilator support [6], [7],raising the possibility at the time of a change in the virulence of the virus.

On 11 May 2009, the first imported human 2009 H1N1 patient was detected in mainland China, and subsequently the first wave of activity occurred from September 2009 to January 2010 during the expected influenza season. Subsequently, from February to December 2010, influenza B and A(H3N2) influenza viruses sequentially predominated in China, but from January through February 2011, 2009 H1N1 was once again the predominant virus in China [8].

The epidemiology of 2009 H1N1 during the pandemic period indicated that the majority of hospitalized and severely ill (intensive care unit [ICU] admission or death) patients occurred in older children and non-elderly adults [9]–[12], in contrast to seasonal influenza infection which affects predominantly children <5 years and the elderly [13]. Similar to seasonal influenza virus infection, underlying risk factors for severe 2009 H1N1 disease include chronic medical conditions and pregnancy. In addition, obesity [10]–[11], [14]–[22], and indigenous/Aboriginal populations [16], [23] have been reported at increased risk of severe 2009 H1N1 disease.

Since seasonal and pandemic influenza viruses undergo constant antigenic drift and may change in virulence, it was not possible to predict the impact of 2009 H1N1 in the post-pandemic period. Therefore, WHO recommended that countries maintain pandemic monitoring systems to detect changes in severity or characteristics of disease and therefore to allow for appropriate targeting of prevention and control and treatment measures such as vaccination, antiviral use, and non-pharmaceutical interventions.

On 30 April 2009, nationwide surveillance for 2009 H1N1 was established through the notifiable infectious disease registry in China, and remained in effect after the pandemic was declared to be over. In this study, we describe the clinical and demographic characteristics of patients hospitalized in China with laboratory-confirmed 2009 H1N1 infection in the post-pandemic period, and examine risk factors for ICU admission and death.

## Materials and Methods

### Patient Definition

A hospitalized case was defined as a patient who was admitted to hospital based on clinical judgment and tested positive for 2009 H1N1 virus by real-time reverse transcription polymerase chain reaction. A severe case was defined as hospitalized patient with laboratory confirmed 2009 H1N1 virus infection who died or who was admitted to the intensive care unit (ICU). A moderately ill case was defined as a hospitalized person who tested positive for 2009 H1N1 but who did not meet the definition of severe case.

### Surveillance System and Data Collection

Beginning on 30 April 2009 all laboratory-confirmed cases with 2009 H1N1 infection nationwide were required to the Chinese Center for Disease Control and Prevention (China CDC) via a web-based reporting system. For each confirmed patients, the basic demographic data including name, age, sex and location were collected. All admitting hospitals were asked to collect more detailed epidemiological and clinical data from hospitalized cases of 2009 H1N1 on a voluntary basis by using one of two methods. Either physicians could conduct a medical chart review and report information through the web-based reporting system to China CDC or hospitals could provide medical records of hospitalized cases to China CDC where two trained clinicians from China CDC performed a medical chart review. A standardized case form was used for data extraction to collect the additional epidemiologic information on demographics, chronic medical conditions, height, weight, pregnancy status, treatment, and outcome of hospitalization. Chronic medical conditions that are associated with higher risk for influenza complications were defined as by the United States Advisory Committee on Immunization Practices [13]. Body mass index (BMI) was calculated for patients as the weight in kilograms divided by the square of height in meters to assess obesity. Obesity was defined according to Chinese criteria as a BMI ≥28 for adults aged ≥18 years [23], or greater than the corresponding cut-off values for children aged 2–17 years [24].

To describe the clinical and demographic characteristics of hospitalized patients and risk factors for severe disease (ICU admission and death) in China during first winter season of post-pandemic, we used data from hospitalized cases from November 2010 through May 2011. We compared the age distribution of hospitalized and fatal 2009 H1N1 cases during the post-pandemic period with hospitalized and fatal cases during the pandemic period (September 2009 to 28 February 2010), which were reported through the same web-based system. To assess the risk of pregnancy and obesity with hospitalization we compared the prevalence of pregnancy among cases with the prevalence of pregnant women in China estimated in the 2008 national census, and compared the prevalence of obesity in cases with that in the latest national nutrition and health survey in 2002. The prevalence of pregnant women was estimated through the 2008 national census data, including the reported number of births, the reported number of induced abortions and estimated number of spontaneous abortions [26], [27]. As this study included data from the National Notifiable Disease Registry system, ethics approval was not required.

### Statistical Analysis

Descriptive statistics including frequency analysis for categorical variables, medians and interquartile ranges (IQRs) for continuous variables were completed. We calculated age-standardized risk ratio (RR) for hospital admission and death. For each age group we compared the proportion of all cases that fell in that age group with what we would expect if the risk of illness was the same across age groups in the general population. A RR above one indicates an excess risk of death or hospitalization due to 2009 H1N1 infection in that age group. Population data by age group were provided from the National Bureau of Statistics of China.

The risk ratios were calculated as follows:

Risk ratio of Hospital admission (RRhosp) = (C_Hospi/_∑C_Hospi_)/(G_i_/∑G_i_)

C_Hospi_: number of hospitalized patients in a given age group

∑C_Hospi:_sum of hospitalized patients in all age groups

G_i_: population in a given age group

∑G_i_: sum of population in all age groups

And risk ratio of death (RRdeath) = (C_Deathi/_∑C_Deathi_)/(G_i_/∑G_i_)

C_Deathi_: number of fatal patients in a given age group

∑C_Deathi_: sum of fatal patients in all age groups.

We assessed risk factors associated with severe illness among non-pregnant patients aged ≥2 years, using univariate analysis and multivariable logistic regression. Univariate analyses with Wilcoxon rank sum test for continuous variables and Chi-square test or Fisher’s exact test for discrete variables were performed with statistical significance defined by an alpha <0.05. Before conducting multivariate analysis, two-way interaction terms between independent variables were tested using the test of homogeneity. A multivariable logistic regression model was used to assess risk factors associated with severe illness among non-pregnant patients ≥2 years of age, including age, gender, chronic medical conditions, obesity and days from symptom onset to hospital admission.

To estimate the effectiveness of early antiviral treatment (within 2 days of symptom onset) among non-pregnant patients aged ≥2 years, only patients who received antiviral treatment and had clinical outcome during study period were involved in this separate analysis, which include antiviral treatment and other same risk factors to the previous model. Odds ratios (ORs) and 95% confidence intervals (CIs) were calculated in the multivariable logistic regression analysis. Data were analyzed with SAS 9.1 (SAS Institute, Cary, NC, U.S.) software.

## Results

From November 2010- May 2011, a total of 8,491 laboratory-confirmed patients from 30 provinces throughout China were reported to the Nationally Notifiable Disease Registry system. Of all 8471 laboratory-confirmed patients, 1,011 patients from 29 provinces were admitted to hospitals (Figure S1). From September 2009 to February 2010, there were 124,319 confirmed cases and 31,610 hospitalized cases.

**Figure 1:**
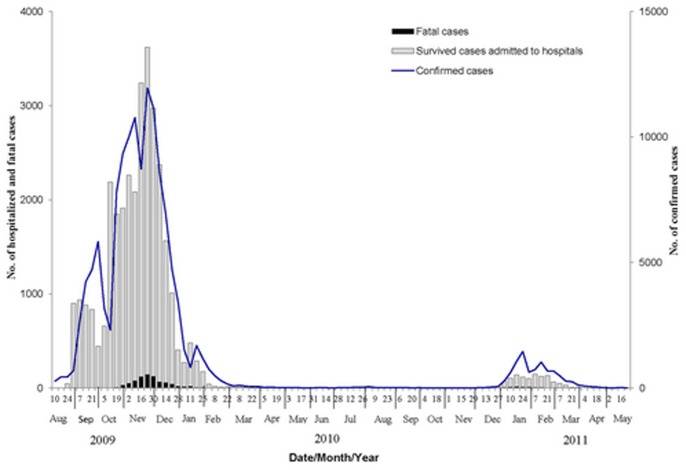
Flow diagram of enrollment of hospitalized cases with confirmed Influenza A(H1N1)pdm09 infection from November 2010 to May 2011.

**Figure 2:**
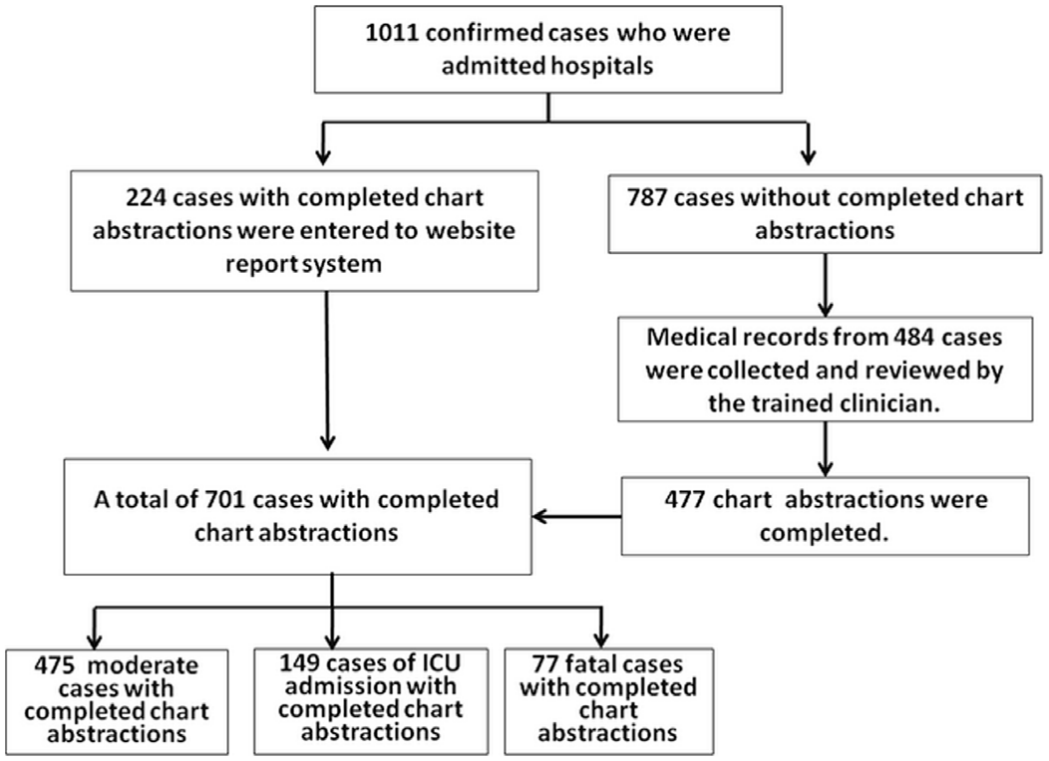
Hospitalized patients with influenza A (H1N1)pdm09 infection by date of symptom onset, China, from September 2009 to May 2011.

Symptom onset dates of patients admitted to hospitals peaked from mid-January 2010 to mid-February 2011, which corresponds to the peak of confirmed cases of 2009 H1N1 from laboratory surveillance data (Figure 1). We obtained completed chart abstractions of 224 hospitalized patients from the reporting system and 477 medical records were sent to China CDC for data extraction. Therefore data from complete chart abstractions were available for a total of 701 hospitalized cases (69.3%) and were included in the analysis. Of these 701 hospitalized cases, 226 were severe cases, comprising including 77 (11.0%) who died, and 149 (21.2%) who were admitted ICU (Figure 2).

### Age Distribution

**Figure 3:**
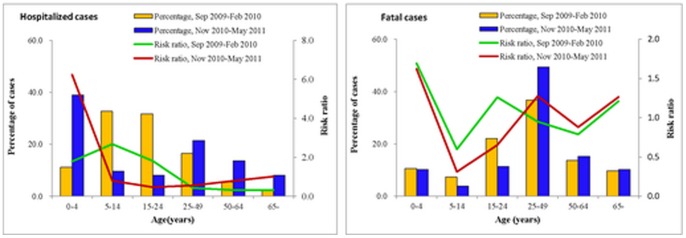
Risk ratio and percentage of hospitalizations and death by age group, China, during the winter season of 2010–2011 and during the 2009 pandemic period.

During the winter season of 2010–2011, 43.2% of hospitalized cases occurred in those older than 25 years of age, whereas only 24.4% occurred during the 2009–2010 pandemic period (p<0.0001) (Figure 3-A). A significantly higher proportion of fatal cases among persons older than 25 years of age during the winter season of 2010–2011was consistently observed, compared to the 2009–2010 pandemic period. (74.7% vs. 60.1%, p<0.01) (Figure 3-B).

The RRs of hospitalization and death of cases as compared to expected in the general population were calculated by age group (Figure 3). The RRs of hospitalization during the winter season of 2010–2011 were 6.2 among people aged 0–4 years and 1.0 among those aged ≥65 years (Figure 3-A). This contrasts with the 2009–2010 pandemic period when the RR for hospital admission was highest in the 5–14 year age group (2.7)(Figure 3-B). The RRs of death above 1 were obtained for three age groups of 0–4, 25–49 and ≥65 years during the winter season of 2010–2011(Figure 3-B). During the 2009–2010 pandemic, RR for death above 1were obtained also in the age groups of 0–4 and ≥65 years, but in the 15–24 years age group rather than 25–49 years age group. The maximum RRs of death were consistently observed in the 0–4 year age group during the 2009–2010 pandemic (1.7) and during the winter season of 2010–2011 (1.6).

### Demographic Characteristics and Underlying Risk Conditions

The median age of the 701 hospitalized cases with chart review during the winter season of 2010–2011 was 22 years (IQR, 3–46 years). The proportion of male patients was 58.6%, which was significantly higher than that among the general Chinese population (51.4%) (p<0.01). Having at least one high risk chronic medical condition was reported in 32.5% of hospitalized cases. The proportion of cases with at least one high-risk chronic medical condition increased with age, from 10.8% among those 5–14 years of age to 86.8% among those 65 years or older (Figure S2). The proportion of patients with at least one chronic medical condition also increased with disease severity, 25.5% among moderately ill patients, 44.3% among patients who admitted to ICU, and 53.3% among cases with fatal outcomes(P<0.0001). The most common chronic medical conditions were cardiovascular disease (11.3%), chronic lung disease (including asthma) (11.1%) and chronic metabolic disease (7.3%). Of all 162 hospitalized patients with known vaccination history, only 5.6% obtained the seasonal influenza vaccination during 2009–2010, and 1.2% vaccinated by 2009 H1N1 monovalent vaccine (Table 1).

**Table 1:** Demographic characteristics, chronic medical condition and treatment of hospitalized cases with influenza A (H1N1)pdm09 infection (N = 701).

Characteristics	Hospitalized Patients with chart review n = 701	Moderately ill Patients n = 475	Severe Patients		
			Subtotal n = 226	Admitted to ICU n = 149	Death n = 77
Age, median (IQR), years	22 (3–46)	14 (2–41)	30 (6–55)	27 (4–55)	39 (25–53)
Male sex	411 (58.6)	271 (57.1)	140 (62.0)	99 (66.4)	41 (53.3)
At least 1 underlying medical condition	228 (32.5)	121 (25.5)	107 (47.4)	66 (44.3)	41 (53.3)
Cardiovascular disease	79 (11.3)	43 (9.1)	36 (15.9)	23 (15.4)	13 (16.9)
Chronic lung disease	78 (11.1)	50 (10.5)	28 (12.4)	24 (14.8)	6 (7.8)
Metabolic disease	51 (7.3)	24 (5.1)	27 (12.0)	18 (12.1)	9 (11.7)
Chronic hepatic disease	39 (5.6)	25 (5.3)	14 (6.2)	8 (5.4)	6 (7.8)
Immunosupression	36 (5.1)	15 (3.2)	21 (9.3)	8 (5.4)	13 (16.9)
Chronic renal disease	28 (4.0)	13 (2.7)	15 (6.6)	10 (6.7)	5 (6.5)
Neurological disease	14 (2.0)	7 (1.5)	7 (3.1)	6 (4.0)	1 (1.3)
Guillainbarre syndrome	2 (0.3)	1 (0.2)	1 (0.4)	1 (0.7)	0 (0)
Vaccination					
Seasonal flu vaccination during 2009–2010	9 (5.6)	7 (9.2)	2 (2.3)	1 (2.2)	1 (2.4)
Pandemic H1N1 vaccination	2 (1.2)	0 (0)	2 (2.2)	1 (2.0)	1 (2.3)
Obesity among non-pregnant patients≥2 yrs of age with known information	71 (17.3)	41 (16.9)	30 (17.9)	21 (19.4)	9 (15.0)
Pregnancy among female patients ofreproductive age (15–49 year-old)	56 (48.7)	30 (45.5)	26 (53.1)	15 (55.6)	11 (50.0)
Days from symptom onset to hospitaladmission, Median(IQR)	3 (1–5)	2 (1–5)	4 (2–7)	3 (1–6)	4 (2–7)
Antiviral treatment	359 (55.9)	191 (43.6)	168 (82.4)	115 (85.8)	53 (75.7)
Antiviral treatment initiated beforehospital admission	8 (2.3)	5 (2.8)	3 (1.7)	3 (2.5)	0 (0)
Antiviral treatment initiation time,median days (IQR)	5 (2–7)	4 (2–7)	5 (3–8)	5.0 (3–7)	7 (4–9)

NOTE. Data are no. (%) of patients, unless otherwise indicated. All patients who died had been admitted to an ICU.

Fifty-six (8.0%) patients were pregnant, including 2 postpartum women who had delivered within 2 weeks from illness onset (Table 1). The median age of pregnant cases was 22 years (IQR, 18–37 yrs), 11(19.6%) had a chronic medical condition. Of all pregnant women, 15 (26.8%) required ICU admission, and 11 (19.6%) died. Approximately, 51.8% of pregnant women were in the second trimester and 26.8% were in the third trimester. The proportion of pregnant women among all hospitalized cases of reproductive-age was significantly higher than the overall proportion of pregnant women among women of reproductive-age in China, from Census data (48.7% vs 3.2%) (p<0.05). The proportion of pregnant cases among severe patients of reproductive-age women was not significantly higher than moderately ill cases of reproductive-age women (53.1% vs 45.5%) (p>0.05).

Of the 411 non-pregnant hospitalized patients ≥2 years of age with known BMI, 71 (17.3%) were obese, and 5 (1.2%) were morbidly obese. The proportion of obesity in this group was significantly higher than the proportion of obesity among the same group of the general Chinese population from the latest national nutrition and health survey in 2002 [25](17.3% vs 7.1%) (p<0.05). Of the 71 obese cases, 21(29.6%) required ICU admission and 9 (12.7%) died. Among obese patients, 40.8% (29/71) had at least one chronic medical condition.

### Treatment

**Figure 4:**
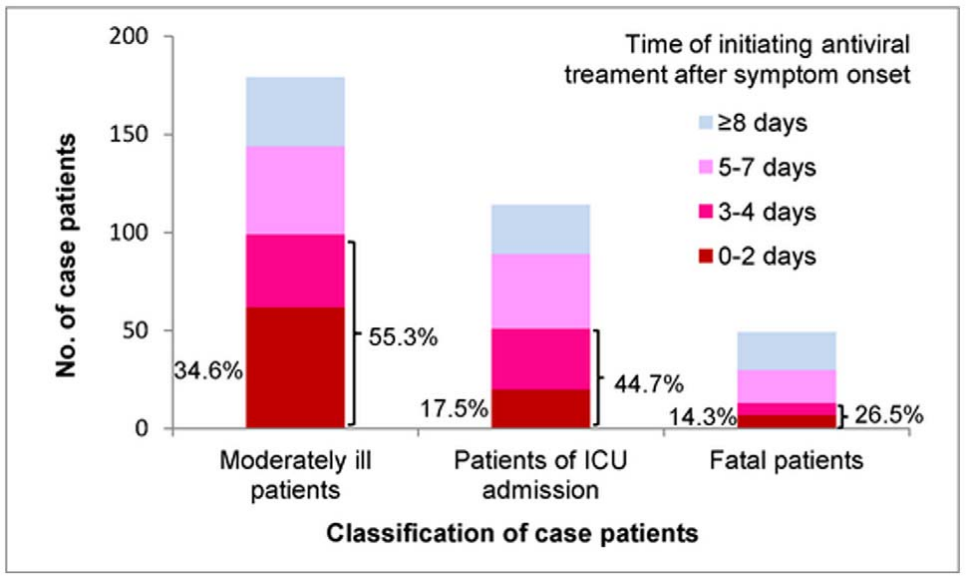
Days from symptom onset to antiviral treatment initiation among Hospitalized cases with influenza A (H1N1)pdm09 infection, China, during the winter season of 2010–2011 (n = 342). Bar labels in the left side of each bar denote percent of hospitalized cases within 2 Days from symptom onset to Antiviral treatment initiation. Bar labels in the right side of each bar denote percent of hospitalized cases within 4 Days from symptom onset to Antiviral treatment initiation.

The median number of days from symptom onset to hospital admission was 3 days (IQR, 1–5 days). Of all hospitalized patients with known information on antiviral treatment, 359 patients (55.9%), including 168 (46.8%) severe patients and 191 (53.2%) moderately ill patients. Antiviral treatment before hospital admission was initiated in only 8 (2.2%) patients. The median number of days between illness onset and initiation of antiviral therapy was 5 days (IQR, 2–7 days). Of 342 patients with known date of antiviral treatment initiation, 26.0% received antiviral treatment within 2 days of onset. The proportion of patients who began antiviral treatment within 2 days from illness onset decreased with increasing disease severity, from 34.6% for moderately ill patients, to 17.5% for those who were admitted to ICU, and to 14.3% for those with fatal outcomes (Figure 4).

### Risk Factors Associated with Severe Illness

A univariate analysis was performed to analyze risk factors between moderately ill cases and severe cases among non-pregnant patients ≥2 years of age (Table 2). The proportion of cases with at least one chronic medical condition was statistically significantly higher for patients with severe disease (53.2%) compared with moderately ill cases (31.0%) (P<0.0001). There was no statistically significant difference in obesity between severe and moderately ill cases (17.9% vs 16.9%). The proportion of severe cases with the delayed hospital admission (≥3 days after onset) (61.6%) was significantly higher than moderately ill cases (44.6%, P<0.001). Among non-pregnant patients aged ≥2 years who used antiviral treatment, the proportion of cases with initiation within 2 days of symptom onset among severe cases was significantly lower than that among moderately ill cases (17.4% vs 34.9%, P<0.001).

**Table 2:** Factors associated with severe illness due to influenza A (H1N1)pdm09 among non-pregnant cases aged ≥2 years.

Characteristics	No. of moderately ill patients (%) n = 342	No. of severe patients (%) n = 188	*Univariate* *		*Multivariate* †	
			*OR (95% CI)*	*p-value*	*aOR (95% CI)*	*p-value*
Male, sex	213 (62.3)	132 (70.2)	1.43 (0.98–2.09)	0.07	1.69 (1.09–2.63)	<0.05
Age, years						
2–17	146 (42.7)	55 (29.3)	Ref		Ref	
18–49	110 (32.2)	65 (34.6)	1.57 (1.01–2.43)	0.68	1.06 (0.63–1.80)	0.80
≥50	86 (25.2)	68 (36.2)	2.10 (1.35–3.27)	<0.01	1.01 (0.56–1.83)	0.93
At least 1 underlying medical condition	106 (31.0)	100 (53.2)	2.53 (1.75–3.65)	<0.0001	2.50 (1.54–4.06)	<0.01
Days from symptom onset to hospital admission						
On symptom day 0–2	189 (55.4)	71 (38.4)	Ref		Ref	
On symptom day ≥3	152 (44.6)	114 (61.6)	2.00 (1.39–2.88)	<0.001	2.00 (1.30–3.04)	<0.01
Days from symptom onset to antiviral treatment initiation‡						
On symptom day 0–2	51 (34.9)	23 (17.4)				
On symptom day 3–4	35 (24.0)	34 (25.8)	2.15 (1.09–4.23)	0.37	1.64 (0.77–3.49)	0.81
On symptom day >5	60 (41.1)	75 (56.8)	2.77 (1.52–5.04)	<0.01	3.12 (1.54–6.35)	<0.01

* The Chi-square test was performed unless otherwise indicated.

† In the multivariate analysis, none of the two-way interaction terms was significant.

‡ Only patients who received antivirus treatment were included in the analysis.

A multivariate analysis was conducted for non-pregnant patients aged ≥2 years (Table 2). Male (OR, 1.69; 95% CI, 1.09–2.63), at least one chronic medical condition (OR, 2.50; 95% CI, 1.54–4.06) and increased time between illness onset and hospital admission (≥3 days) (OR, 2.00; 95% CI, 1.30–3.04) were independent risk factors for severe illness among non-pregnant cases ≥2 years of age.

In a separate model including antiviral treatment among non-pregnant cases who were treated with antiviral therapy, initiating antiviral treatment ≥5 days after symptom onset (OR, 3.12; 95% CI, 1.54–6.35) was associated with the severe illness compared with antiviral treatment initiation within 2 days from symptom onset, but initiating antiviral treatment 3–4 days from symptom onset (OR, 1.64; 95% CI, 0.77–3.49) was not statistically associated with severity.

## Discussion

In this study, we observed differences in the age distribution and risk factors for severe illness between the first winter season of post-pandemic period and the 2009–2010 pandemic period. A shift to older ages in the age distribution of hospitalized and fatal patients were observed during the winter season of 2010–2011, which was consistent with data from the United Kingdom, Greece and Taiwan [28]–[30]. During the winter season of 2010–2011, children aged 0–4 years and adults aged 65 years or older had the highest risk ratios of hospitalization, while people under 25 years of age had the highest risks of hospitalization (peak 5–14 years) during the 2009–2010 pandemic. During the winter season of 2010–2011, risk ratios of hospitalization in the 5–14 and 15–24 years age groups declined, compared with the 0–4 years age group. The change of higher risk age groups might be explained by highest immunity to 2009 H1N1 in the 5–14 and 15–24 years age groups after experiencing the pandemic wave which was reported from serological study in China and other countries [31]–[32]. The high risk of death due to 2009 H1N1 were consistently observed among children of 0–4 years and older adults aged 65 years or older during the winter season of 2010–2011 and the 2009–2010 pandemic. For children aged 0–4 years, the greater risk for hospitalization than for death with 2009 H1N1 infection may have resulted from a lower threshold for hospital admission and therefore inflate the calculated Risk Ratio compared to other age groups. During the 2009–2010 pandemic, studies in several countries reported that obesity was associated with severe or fatal 2009 H1N1 virus disease [14] [19]. Although our study indicated the proportion of obesity among hospitalized patients was significantly higher than the general Chinese population, obesity among hospitalized cases was not a statistically significant risk factor for severe complications from 2009 H1N1 virus infection during the winter season of 2010–2011. This is in contrast to a previously published study in China during the 2009–2010 pandemic [11]. The absence of an association between obesity and severe outcomes may be explained by the higher proportion (40.8%) of chronic medical conditions among obese patients who were admitted hospitals in our study, compared to the previously published study in China (24%). Additionally, the number of obese patients in this study was small limiting statistical power to detect an association with severe outcomes.

Consistent with studies describing seasonal influenza and other studies about the 2009 H1N1 pandemic [10]–[11], [13]–[22], the presence of at least one chronic medical condition was associated with 2009 H1N1 severe illness. In our study, a higher proportion of severe cases had at least one underlying medical condition (47.4%) was observed compared to the previous study conducted during the pandemic period in China (33%) [11].

Consistent with the previous studies of seasonal influenza and 2009 H1N1 pandemic, our results reaffirmed that early initiation of oseltamivir treatment may reduce the risk of influenza-associated complications. However, our study observed lower usage of antiviral therapy (55.9%), compared to the previously published study from the pandemic period in China (76%) [11]. The proportion of antiviral treatment within 2 days from symptom onset in our study was low (26.0%), but higher than the study of hospitalized cases (17%) in China during the pandemic period [11]. Some reasons for the delay in treatment initiation included waiting for laboratory confirmation of 2009 H1N1, delays in healthcare presentation, or the reduced awareness of antiviral treatment. Although antiviral treatment is accessible at different healthcare settings, our study showed only a small proportion of patients received antiviral treatment before admission to the hospital. According to current Chinese influenza surveillance data, nearly all 2009 H1N1, H3N2 and B virus strains tested were susceptible to neuraminidase inhibitors (oseltamivir and zanamivir) [8]. People with underlying medical conditions and other possible risk factors for severe disease from influenza virus infection should be educated to seek treatment promptly after onset of an influenza-like illness to ensure that antiviral treatment if appropriate is initiated in a timely fashion. Recommendations to healthcare providers should suggest providing early empiric treatment with appropriate influenza antiviral medications to suspected cases of influenza virus infection, both in outpatient settings and inpatient wards, especially to those patients who may be at higher risk of influenza virus infection complications.

Our findings indicated that male patients were more likely to develop severe illness, which was consistent with the previously published study in China during the 2009–2010 pandemic period [11]. Nevertheless, a global pooled analysis showed that men were approximately half of all hospitalized, ICU-admitted, and fatal cases^10.^ It was also observed in studies from South Korea, that mean had a significantly higher proportion of pneumonia [33]–[34]. The association between men and severe illness of 2009 H1N1 may reflect different behaviors, underlying medical conditions, susceptibility to 2009 H1N1 virus infection and other unrecognized risk factors for severe illness among men.

Our study had a number of limitations that should be noted. The reported hospitalized patients in this study only represented a portion of the total number of actual hospitalized patients with 2009 H1N1 infection due to limitations of the clinical surveillance system in capturing individuals who seek medical care at hospitals and obtain laboratory test. There is a decrease of the numbers of influenza-confirmed patients and hospitalized patients during the winter of 2010–2011 compared to 2009–2010 pandemic period. This decline may due to more under-reporting (compared to a more strengthened surveillance during pandemic period) or due to a high immunity level against 2009 H1N1 in the population. Some of the associations with age groups may have been due to underreporting or overreporting of cases in any one group. Chart abstractions or submission of medical records to China CDC were performed voluntarily, rather than systematically which reflects the willingness and capacity of physicians to perform them. In this case series, the high death to hospitalization ratio (7.8%) may be a result of case referral bias in this voluntary case review/submission process. Our study may be biased towards older adults in the analysis of risk factors because patients who had a chart review were older, compared with those patients without chart review. Influenza vaccine information of many hospitalized cases were missing in this study because vaccine history is not a required data in medical records in most of hospitals in China. Thus, our findings should be interpreted with caution because of the retrospective study design, selection bias and small sample size.

Despite our study limitations, we observed some important trends in severe infection with 2009 H1N1 virus infection. This study indicated age groups at higher risk of hospitalization during the immediate post-pandemic period were changing, compared with those during the 2009–2010 pandemic. During the winter season of 2010–2011, children under 5 years had the highest risk of hospitalization and death associated with 2009 H1N1 infection. Additionally, a decline of risk of hospitalization among people aged 5–24 years and a shift to older age for fatal patients was detected. The relative risk of hospitalization and death among people older than 64 years increased. Consistent with seasonal influenza and the 2009–2010 pandemic period, chronic medical conditions are important risk factors for severe disease during the winter season of 2010–2011. This study demonstrated the benefit of maintenance of severe patients surveillance to determine changes in the epidemiology of 2009 H1N1 infection after the pandemic period, and contributing to recommendations to target groups for influenza prevention and control interventions.

## Supporting Information

Figure S1 **Geographical distribution of all hospitalized cases reported to China CDC, China, from November 2010 to May 2011.**
(TIF)

Figure S2 **Age distribution of hospitalized caseswith and without chronic medical conditions, the 2010–2011 winter season.** Bar labels denote percent of hospitalized cases with chronic medical conditions in each age group.
(TIF)
