# Supplementary figures and images for: Characteristics of Hospitalized Cases with Influenza A (H1N1)pdm09 Infection during First Winter Season of Post-Pandemic in China
Source: PLoS One. 2013 Feb 5;8(2):e55016. doi: 10.1371/journal.pone.0055016 (PMC3564919; doi:10.1371/journal.pone.0055016)

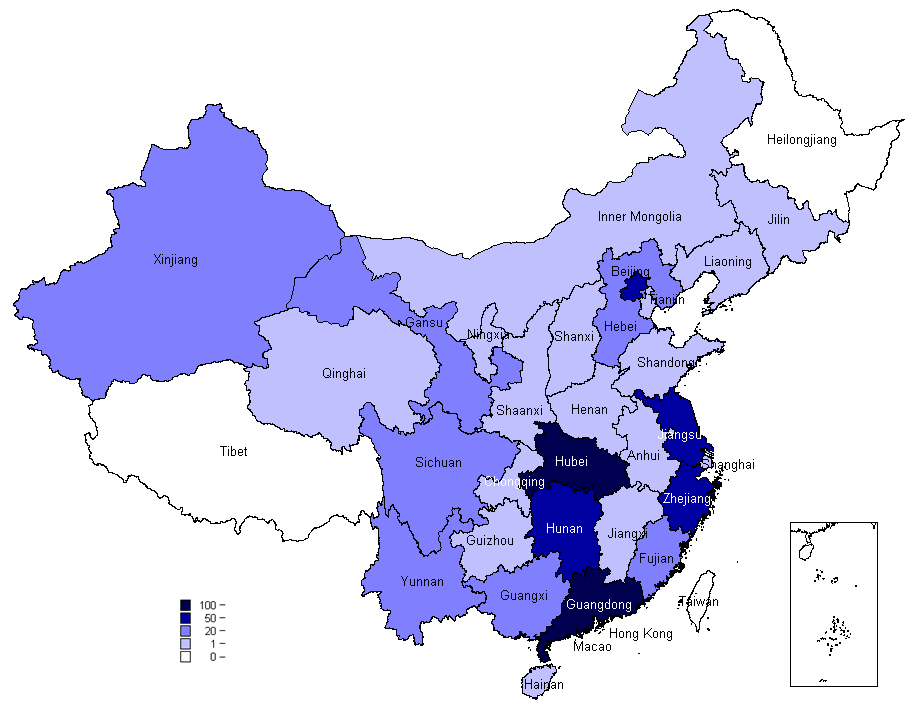

Supplement: Figure S1 — Geographical distribution of all hospitalized cases reported to China CDC, China, from November 2010 to May 2011. (TIF) [file pone.0055016.s001.tif]

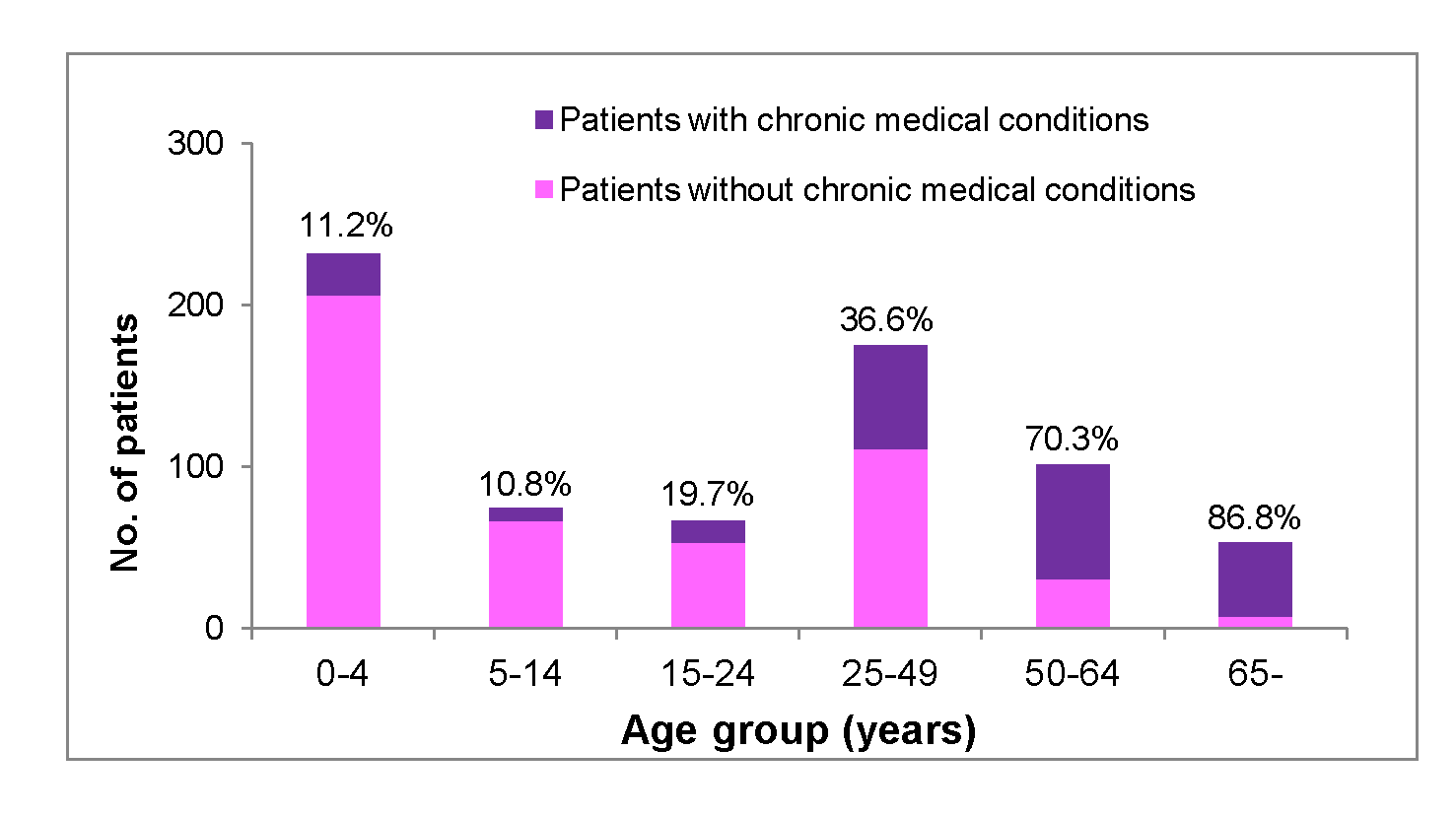

Supplement: Figure S2 — Age distribution of hospitalized caseswith and without chronic medical conditions, the 2010–2011 winter season. Bar labels denote percent of hospitalized cases with chronic medical conditions in each age group. (TIF) [file pone.0055016.s002.tif]
